# Future of Decellularized Dental Pulp Matrix in Regenerative Endodontics

**DOI:** 10.1055/s-0041-1741012

**Published:** 2022-01-06

**Authors:** Zohaib Khurshid, Ahmed Jamil Ahmed Alnaim, Ahmed Abdulhakim Ahmed Alhashim, Eisha Imran, Necdet Adanir

**Affiliations:** 1 Department of Prosthodontics and Dental Implantology, College of Dentistry, King Faisal University, Al-Ahsa, Kingdom of Saudi Arabia; 2 Dental Complex, College of Dentistry, King Faisal University, Al-Ahsa, Kingdom of Saudi Arabia; 3 Department of Dental Materials, Islamabad Medical and Dental College, Islamabad, Pakistan; 4 Department of Restorative Dentistry, College of Dentistry, King Faisal University, Al-Ahsa, Kingdom of Saudi Arabia

**Keywords:** tooth, dental pulp tissue, root canal treatment, decellularization, regenerative endodontics

## Abstract

With the advancements in tissue engineering, the repair and regeneration of oral/dental tissue are becoming possible and productive. Due to periodontal diseases, the tooth loses bone support resulting in tooth loss, but bone grafting stabilizes with new bone. It is seen that due to the progression of dental caries, pulp damage happens, and the vitality of the tooth is compromised. The current theme of dental pulp regeneration through biological and synthetic scaffolds, is becoming a potential therapy for pulp revitalization.

## Introduction

Dental pulp can be considered as the heart of a tooth. Its composition is complex and consists of organic and inorganic material, including connective tissue, blood vessels, and a cluster of different types of cells. 1 Dental pulp has a significant role in the integrity of the tooth, tooth vitality, providing the tooth with nutrients through blood vessels, and aiding the formation of dentin in the case of tissue damage through the formation of odontoblast cells. 2 The pulp is richly innervated with different types of nerves, including sensory, sympathetic, and parasympathetic nerve fibers. The sensory fibers have a significant role as they act as an alarm system if there is an inflammation or any pulp damage is present. Pain is mediated mainly with two different sensory fibers: A-delta fibers, which are myelinated and moderately fast in conduction that convey sharp pain and cold sensation, and C-fibers, which are nonmyelinated, slow in conduction, and convey dull pain. 3

Dental caries represent one of the principal challenges to the health of the dental pulp, although its treatment may well exacerbate the challenge. 4 A fundamental consideration in pulp protection is recognizing that infection is a crucial driver of inflammation, which often determines the outcomes for pulp survival. Thus, the pulp always is likely to be inflamed when bacteria from dental caries are present, and their control should be a significant feature of any treatment planning. Regarding the conventional intervention to treat damaged pulp tissue, some drawbacks might affect the prognosis of the tooth even if the root canal treatment is done ideally. The tooth is mainly affected structurally as the tooth is eliminated of living tissues, debrided of necrotic dentin, and the canal spaces become widened, thus leaving the tooth dehydrated and fragile. It is important for the pulp to be healthy to perform its functions such as nutrient supply to the teeth, dentine formation, sensing, defense, and other physiological functions. The future of endodontics aims to ensure pulp vitalization by pulp tissue regeneration, using xenograft-derived stem cells to accomplish cell proliferation that could replace the diseased pulp tissue with healthy ones, regaining the structural and biological integrity of the pulp and tooth structure. 3 5 6

Maintenance of pulp vitality should always be the goal in treatment planning, and considerable interest is developing in the concept of regenerative endodontics for complete or partial pulp tissue regeneration. 7 A new pathway has been opened due to advancement in regenerative medicine by transplanting stem cells along with the growth factors and a biological scaffold into the prepared pulp cavity. These stem cells can proliferate and differentiate into various cells in the pulp to achieve functional pulp regeneration. 1 3 5 Basically, regenerative endodontics has been pioneered by the experimental studies of Nygaard-Ostby and Hjortdal, involving induced bleeding from the periapical tissues into the chemo-mechanically debrided canal space of teeth, which was partly filled with root filling. 8

Regeneration is a process by which altered tissues are entirely replaced by tissues native to their original architecture and function. 9 It is based on the concept of tissue engineering technology that regenerates the dentine pulp complex in the canal space of immature permanent teeth, which could either be damaged by caries or trauma, and restores development of the arrested tooth root.

## Decellularization of Dental Pulp Tissue

**Fig. 1:**
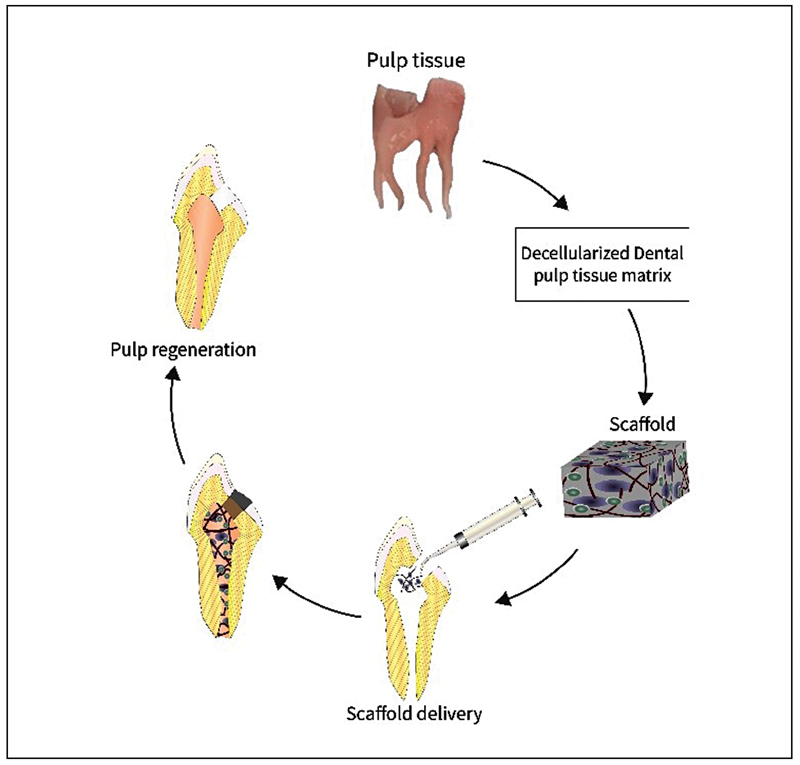
Diagrammatic representation of the preparation of dental pulp tissues for regenerative dentistry.

Decellularization can be described as a procedure that aims to remove cellular contents of a tissue, leaving the extracellular matrix (ECM) free of antigens that could produce an inflammatory reaction, thus reducing the risk of spread of disease as well as leaving the tissue in its original three-dimensional biostructure. 10 The process of decellularization is complex and can be achieved in different ways; it can be done mechanically, chemically/enzymatically, or by combining these methods. This is done to preserve the nanostructure environment of fibrous and adhesive proteins, which will aid in cell anchorage and regulate cellular activities in the future. By doing so, we can achieve local resident cell support, which can be a big step in pulp/dentine tissue engineering. In Fig. 1 , diagrammatic representation of the decellularization of dental pulp tissues for regenerative endodontics has been provided. There are many procedures and protocols that are reported for the decellularization of the dental pulp tissues, as mentioned in Table 1 .

**Table 1:** Decellularized dental pulp tissue method of preparation, testing, and outcomes of the studies

Author and year	Source of sample	Preparation/Extraction method	Evaluation method	Result and outcomes
Bakhtiar et al (2021) 11	Bovine dental pulp	• Fresh dental pulp tissue extracted and treated with series of chemical treatment • Seven different protocols were designed for decellularization	• Histologic analysis, DNA content analysis, immunofluorescence, and animal immunogenicity testing	• All designed protocols of decellularization showed the reduction in DNA content. • Twelve hours of EDTA/trypsin treatment and 1 hour of Triton X-100 treatment with no SDS showed good results for using bovine dental pulp xenograft for endodontic regeneration
Bakhtiar et al (2020) 1	Bovine dental pulp	• Fresh pulp tissue extracted from bovine teeth and washed • Treated with trypsin, EDTA, and SDS • Lyophilized and stored at −20°C	• Physiochemical analyses, cell viability, RT-PCR • Animal study	• Scaffolds revealed low immunological response • Angiogenesis enhanced • Biodegradability decreased • Biocompatibility increased
Lee et al (2020) 12	Human dental pulp	• Pulp extracted and washed with dH _2_ O for 1 hour • Pulp decellularized by using 1% Triton X-100 and 0.1% ammonium hydroxide on a shaker at 4°C. • Lyophilized and sterilized using ethylene oxide gas	• SEM, Micro-CT, EDX • Biochemical, biomechanical • VEGF measurement and histological analyses	• EDX revealed the high mineralization in the pulp matrix than seen in calvarial bone • Traces of blood vessels observed in new bone regenerated with DPM
Li et al (2020) 13	Human dental pulp	• Extracted human dental pulp rinsed with heparinized PBS, followed by the decellularization process • The pulp matrix is then lyophilized, powdered, and stored at −40°C	• Proteomic analysis, cell culture, cellular behavioral assessment, differential induction, RT-PCR, and western blotting	• Hydrogel-derived matrix from human dental pulp showed cell proliferation, migration, and multidirectional differentiation
Alqahtani et al (2018) 9	Swine dental pulp	• Pulp tissue extirpated, followed by decellularization, tissue characterization, and sterilization	• ELISA, SEM, Micro-CT, histology, Immunohistochemistry	• Decellularized pulp ECM proved the structural and functional integrity for providing optimum microenvironment for dental pulp regeneration
Song et al (2017) 14	Human dental pulp	• After removal of pulp tissues, it was stored in cold Hanks' balanced salt solution and then decellularized	• DNA analysis, hydroxyproline quantification, protein extraction, western blot analysis, SEM, RT-PCR	• Successful decellularization of human dental pulp tissues • Biocompatible scaffolds for the proliferation and differentiation of stem cells from apical papilla
Hu et al (2017) 10	Swine dental pulp	• Extracted pulp tissue rinsed with heparinized PBS for 15 minutes and then placed in SDS for 32 hours • After washing, it was treated with 1% Triton X-100 and then final decellularized product sterilized for further investigation	• SEM, histological and immunostaining analysis	The result shows that decellularized swine dental pulp maintains ECM components that favor stem cell proliferation and differentiation, which represents a suitable bioscaffold for improving clinical outcomes as well as functions of teeth with dental pulp diseases
Matoug-Elwerfelli et al (2017) 15	Human dental pulp	• Pulp tissues treated with hypotonic Tris-buffer, EDTA, and aprotinin at 4°C	• Histological, immunohistochemical method, SEM, DNA quantification assay • Cell viability testing	• The results showed that assessment of decellularized tissues revealed an acellular matrix with preservation of native tissue histoarchitecture and composition • No evidence of cytotoxicity seen in decellularized tissues

Abbreviations: DNA, deoxyribonucleic acid; DPM, dental pulp matrix; ECM, extracellular matrix; EDTA, ethylenediamine tetraacetic acid; EDX, energy-dispersive X-ray; ELISA, enzyme-linked immunoassay; Micro-CT, microcomputed tomography; PBS, phosphate buffered saline; RT-PCR, reverse transcription polymerase chain reaction; SDS, sodium dodecyl sulfate; SEM, scanning electron microscopy; VEGF, vascular endothelial growth factor.

It is also seen that regeneration of a pulp structure is usually unlikely to be successful. Still, if basic principles of tissue engineering are implemented within any regenerative endodontic procedures, it may seem to work. Also, reviews of regenerative endodontic procedures, known as revitalization procedures, have concluded that the outcomes are very unpredictable. 16 In Table 1 , description of the decellularization process reported by the researchers for the extraction of bioactive ingredients from dental pulp tissues of different sources has been given. 11 12 13 14 15

Recent discoveries in endodontic dentistry have proven that the human dental pulp is capable of regeneration by using the methods of decellularization by making use of the dental pulp as a scaffold for the potential regeneration process. 9 The human dental pulp is known to contain a variety of cells, primarily identified as stem cells that have a potential to induce differentiation to multiple classes of different cells, including osteoblasts, odontoblasts, neural cells, and adipocytes. Decellularized extracellular matrix (dECM) derived from either human or animal tissues has been considered as a possible scaffolding medium for tissue regeneration in present studies. 11 A study has proven that dECM extracted from dental pulps can stimulate differentiation in periodontal ligament stem cells and in bone marrow, stromal cells, without any other external contributing factors. Another study, done by Song et al, demonstrated that the process of proliferation of dental pulp stem cells from the apical papilla and their differentiation to odontoblastic cells was assisted by the decellularized human dental pulp. 14

**Fig. 2:**
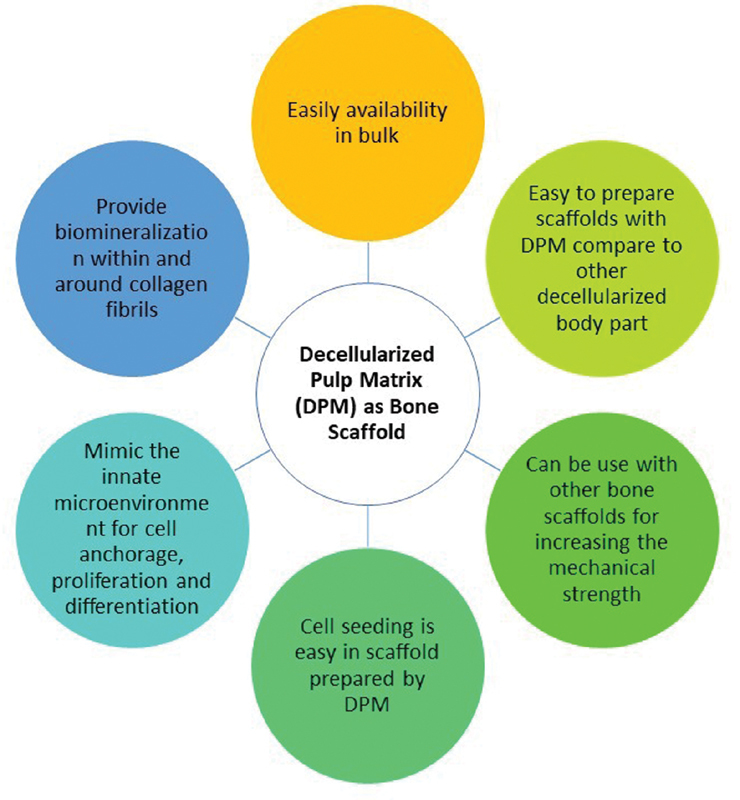
Advantages of dental pulp matrix as a scaffold for bone tissue engineering.

On the other hand, they also found that the scaffold was permanently fixed in shape. It was difficult to insert into narrow and oblique canals of the roots, restricting their potential advantage in clinical application, thus resulting in limited success. Until now, there are no well-founded scaffolds that can mimic the complex ECM of the dental pulp. Therefore, it results in a challenge in producing a regenerative microenvironment for stem cell differentiation. Fig. 2 discusses the benefits of using decellularized dental pulp tissue matrix as a bioscaffold.

## Conclusion

The dental pulp is a unique tissue that contains specialized resident cells, stem cells, and immune cells that contribute to the pulp's defense strategy. It is seen that dental pulp has a substantial ECM that helps maintain the tissue's integrity. Due to their broad activity against a range of pathogens, it is known that novel peptide-based therapeutics are more effective against polymicrobial infections such as endodontic infections. It is believed that it is possible to develop a decellularized biocompatible biological scaffold containing the native ECM structural components that are required for tissue-specific regeneration. It is also seen that regenerative endodontics opens exciting opportunities for the preservation of pulp vitality, which underwent episodes of trauma and disease, and can encourage continued root maturation of immature permanent teeth with necrotic pulp. Overall, it is seen that the possibility to decellularize healthy dental pulp does open new horizons in regenerative dentistry as these decellularized tissues could help in serving as natural scaffolds.

## References

[1] Bakhtiar H Pezeshki-Modaress M Kiaipour Z Pulp ECM-derived macroporous scaffolds for stimulation of dental-pulp regeneration process Dent Mater 2020 36 01 76 87 31735424 10.1016/j.dental.2019.10.011

[2] Zhang X Li H Sun J Cell-derived micro-environment helps dental pulp stem cells promote dental pulp regeneration Cell Prolif 2017 50 05 e12361 10.1111/cpr.12361 28741725 PMC6529091

[3] Smith A J Reflections and future visions for pulp biology research J Endod 2020 46(9S):S42 S45 32950194 10.1016/j.joen.2020.06.023

[4] Paduano F Marrelli M White L J Shakesheff K M Tatullo M Odontogenic differentiation of human dental pulp stem cells on hydrogel scaffolds derived from decellularized bone extracellular matrix and collagen type I PLoS One 2016 11 02 e0148225 10.1371/journal.pone.0148225 26882351 PMC4755593

[5] Simon S Smith A J Regenerative endodontics Br Dent J 2014 216 06 E13 10.1038/sj.bdj.2014.243 24651364

[6] Zafar M S Khurshid Z Almas K Oral tissue engineering progress and challenges Tissue Eng Regen Med 2015 12 06 387 397

[7] Kim S G Malek M Sigurdsson A Lin L M Kahler B Regenerative endodontics: a comprehensive review Int Endod J 2018 51 12 1367 1388 29777616 10.1111/iej.12954

[8] Nygaard-Ostby B Hjortdal O Tissue formation in the root canal following pulp removal Scand J Dent Res 1971 79 05 333 349 5315973 10.1111/j.1600-0722.1971.tb02019.x

[9] Alqahtani Q Zaky S H Patil A Beniash E Ray H Sfeir C Decellularized swine dental pulp tissue for regenerative root canal therapy J Dent Res 2018 97 13 1460 1467 30067420 10.1177/0022034518785124

[10] Hu L Gao Z Xu J Decellularized swine dental pulp as a bioscaffold for pulp regeneration BioMed Res Int 2017 2017 9.342714E6 10.1155/2017/9342714 PMC5745671 29387727

[11] Bakhtiar H Rajabi S Pezeshki-Modaress M Optimizing methods for bovine dental pulp decellularization J Endod 2021 47 01 62 68 33049226 10.1016/j.joen.2020.08.027

[12] Lee D J Miguez P Kwon J Decellularized pulp matrix as scaffold for mesenchymal stem cell mediated bone regeneration J Tissue Eng 2020 11 2.041731420981672E15 10.1177/20417314209816722 PMC7750895 33414903

[13] Li J Rao Z Zhao Y A decellularized matrix hydrogel derived from human dental pulp promotes dental pulp stem cell proliferation, migration, and induced multidirectional differentiation in vitro J Endod 2020 46 10 1438 1.447E8 32679242 10.1016/j.joen.2020.07.008

[14] Song J S Takimoto K Jeon M Vadakekalam J Ruparel N B Diogenes A Decellularized human dental pulp as a scaffold for regenerative endodontics J Dent Res 2017 96 06 640 646 28196330 10.1177/0022034517693606

[15] Matoug-Elwerfelli M Duggal M S Nazzal H Esteves F Raïf E A biocompatible decellularized pulp scaffold for regenerative endodontics Int Endod J 2018 51 06 663 673 29197101 10.1111/iej.12882

[16] Zhang W Vazquez B Oreadi D Yelick P C Decellularized tooth bud scaffolds for tooth regeneration J Dent Res 2017 96 05 516 523 28118552 10.1177/0022034516689082 PMC5453498

